# Prenatal and Postpartum Care Experiences Among Black Birthing People in the United States: An Integrative Review

**DOI:** 10.1111/jmwh.13705

**Published:** 2024-11-13

**Authors:** Laura M. Segovia, Emily Neiman, Shannon L. Gillespie, McKenzie K. Jancsura, Cindy M. Anderson

**Affiliations:** ^1^ The Ohio State University, College of Nursing Columbus Ohio

**Keywords:** African American or Black, care, collaboration, experience, postpartum, prenatal

## Abstract

**Introduction:**

Among Black birthing people, high‐quality, respectful care throughout pregnancy and postpartum is hindered by distrust, racial discrimination, and unsatisfactory care. The purpose of this integrative review was to examine prenatal and postpartum care experiences among Black birthing people in the United States.

**Methods:**

A literature search, spanning from inception through October 6, 2022, across 4 research databases, used a combination of keywords to capture reports on care experiences among Black birthing people. We included quantitative and qualitative studies in the United States with people who self‐identified as Black or African American and reported prenatal or postpartum health care experiences. Intrapartum experiences were excluded. All studies were evaluated with the Mixed‐Methods Appraisal Tool, National Institutes of Health Study Quality Assessment tool, or Joanna Briggs Institute critical appraisal checklist. Data were analyzed and synthesized using the Joanna Briggs Institute convergent integrated approach to incorporate quantitative and qualitative research.

**Results:**

A total of 16 studies published over 27 years met the inclusion criteria. All studies examined the health care experiences of Black birthing people during prenatal or postpartum care. None of the studies focused solely on postpartum care experiences. The 2 most prominent themes were models of care and patient‐provider interactions, encompassing both positive and negative experiences. Positive care experiences included collaborative patient‐provider interactions, continuity of care, and culturally centered care. Adverse experiences were more frequently noted and involved discriminatory treatment during patient‐provider interactions, fragmented care models, and a lack of cultural awareness.

**Discussion:**

Black birthing people in the United States report some positive but more negative health care experiences during pregnancy and postpartum care, which may play an important role in health inequities. Promoting prenatal and postpartum care models that provide continuity and are high‐quality, collaborative, and culturally centered were identified as high‐priority targets to foster patient safety and improve clinical outcomes.

## INTRODUCTION

Black birthing people disproportionately experience higher maternal mortality rates when compared with their White or Hispanic peers (69.9, 26.6, and 28 deaths per 100,000 birthing people, respectively, in 2021).^1^ Additionally, the rates endure among Black birthing people, spanning across age groups, socioeconomic statuses, and geographic regions.^1^, ^2^, ^3^ Approximately 80% of pregnancy‐related maternal deaths are due to preventable causes (ie, preeclampsia, infection, hemorrhage),^4^ and Black birthing people are 2 to 3 times more likely to die from a preventable cause than White birthing people.^5^

**QUICK POINTS**

✦ Examining health care experiences of Black birthing people during pregnancy and postpartum is a crucial starting point to pinpoint priority targets that promote patient safety and improve clinical outcomes.
✦ Positive and negative health care experiences were reported during prenatal and postpartum care, although negative experiences were more commonly described.
✦ This review systematically identifies targets for health care professionals and researchers to improve care experiences among Black birthing people in the United States.

The reasons for higher mortality rates among Black birthing people are complex. However, growing evidence suggests that systemic and structural racism and the resultant consequence of racism and discrimination across the life course are at the heart of the issue.^6^ The complex history of race and ethnicity in the United States can shape an individual's health care experience in multiple ways, contributing to disparate outcomes across races and ethnicities.^7^ For example, the World Health Organization promotes high‐quality, respectful, and dignified care throughout pregnancy and postpartum.^8^ However, prenatal care among Black birthing people has been characterized by distrust,^9^ blatant racial discrimination,^10^, ^11^ and unsatisfactory care.^12^

It is also important to note that 83% of maternal deaths occur outside of childbirth,^13^ with prenatal and postpartum care experiences being critical drivers of perinatal health that have received increased attention. Prenatal and postpartum care provides an important opportunity for risk assessment, enabling providers to prevent, identify, and manage conditions known to contribute to maternal morbidity and mortality.^14^ A number of factors are known to influence prenatal and postpartum care, including an inclusive and inviting care setting,^8^, ^15^ positive health care interactions,^16^ high‐quality health care communication, provider awareness of patient expectations and rights, the delivery of clinical care with dignity and respect, and access to supportive resources.^8^ However, such principles are not always enacted, and racial and cultural differences in attitudes, biases, beliefs, values, and experiences shape the care experience.^17^

Despite the pervasive systemic racism^18^ and discrimination that affects the quality of health care received in the United States,^19^ literature on the lived experience of prenatal and postpartum care among Black birthing people has not been systematically reviewed. Thus, the aim of this review was to examine prenatal and postpartum care experiences among Black birthing people in the United States. The findings may help illuminate existing gaps in care experiences and serve as an important starting point to identify and promote high‐quality, respectful, and culturally congruent prenatal and postpartum care targeted toward the needs of Black birthing people in the United States.

## METHODS

### Information Sources and Search Strategy

The integrative review was prospectively registered on PROSPERO (CRD42022362185, September 22, 2022), and the results were reported according to the Preferred Reporting Items for Systematic Reviews and Meta‐Analysis guidelines (Supporting Information: Table S1). Eligible studies were identified by systematically searching electronic databases (CINAHL, Scopus, Embase, and PsycINFO). The search was conducted using the keywords *prenatal*, *postpartum*, *African American* or *Black*, *care*, and *experience* without a time constraint to be as inclusive as possible (Supporting Information: Table S2). The search was completed on October 6, 2022. Search terms were combined to identify reports on Black birthing people's prenatal and/or postpartum care experiences in the United States. Only articles in English, that were peer reviewed, and with full‐text availability were included. Additionally, the reference lists of the included articles were hand‐searched to capture any additional applicable studies for inclusion.

### Eligibility Criteria and Selection Process

We included quantitative, qualitative, and mixed‐method studies (1) with patients who self‐identified as African American or Black, (2) with a focus on prenatal and postpartum health care experiences, (3) and that took place in the United States. Studies that reported on more than one race or ethnicity must have stratified the results by race and ethnicity to be included. We defined prenatal care as the outpatient care of a pregnant person from conception to labor onset.^14^ Postpartum care was defined as outpatient care occurring from one week to one year postpartum focused on birth, pregnancy, or routine aftercare.^20^ Excluded studies involved (1) case reports, review articles, commentaries, letters, editorials, or conference abstracts; (2) intervention studies without baseline data or a control group; and (3) studies of intrapartum or antepartum inpatient care (ie, in‐patient monitoring, emergency room, triage, labor and birth) only.

Each article was imported into the online platform Covidence, and duplicate studies were omitted. Two authors (C.M.A. and E.N.) independently reviewed the title and abstract for full‐text review. Two authors (M.K.J. and L.M.S.) independently conducted a full‐text review using the inclusion and exclusion criteria. If a disagreement occurred during article selection and could not be resolved with discussion, a senior team member (C.M.A. or S.L.G.) decided on inclusion.

### Quality Appraisal

Two reviewers (S.L.G. and L.M.S.) independently evaluated the risk of bias. The tool used to assess the risk of bias was determined based on the study design. The Mixed‐Methods Appraisal Tool (MMAT, 2018 version) was used to establish the methodological quality of the mixed‐method study. The National Institutes of Health Study Quality Assessment tool was used for observational cohort and cross‐sectional studies. The Joanna Brigg's Institute (JBI) critical appraisal checklist for qualitative research was used for qualitative studies. The overall quality appraisal for each study was assigned as high (>0.80), moderate (0.51‐0.79), or poor (<0.50). Any disagreements were resolved by discussion between the 2 reviewers.

### Data Extraction and Synthesis

Two authors (M.K.J. and L.M.S.) independently extracted data using the online platform Qualtrics. Pilot testing and amendments were made to the extraction form before beginning the full review. Definitions of prenatal and postpartum care experiences were reviewed for clarification to ensure the reliability of the codification process. For each study, the title, author and date, study design, sample size, timing of care (ie, pregnancy, postpartum, or both), care provider type (eg, physician, certified nurse‐midwife, lactation consultant), care setting, inclusion and exclusion criteria, methodology for measurement of care experiences, and a summary of findings were entered into the Qualtrics form. Any disagreements were resolved by discussion between the 2 reviewers.

The JBI framework for mixed‐methods systematic review using the convergent integrated approach was chosen to incorporate both quantitative and qualitative research findings. Converting quantitative data to a qualitative format, or *qualitzing*, is less likely to be error prone than attributing numerical values to qualitative data.^21^ Qualitzing data transforms the quantitative values into themes, categories, or narratives.^21^ Thomas and Harden's (2008) thematic synthesis is the most common approach for this integration.^22^ Two authors (E.N. and L.M.S.) developed the first version of the codebook created from the research question. The codebook was initially developed after both authors’ independent coding was compared and reviewed. A final codebook version was expanded after all authors evaluated, analyzed, and discussed the codes. Based on the data from the codes, ideas that reflected similar concepts were grouped into broad themes and subthemes. Finally, the results of the qualitative studies were compared to those of the quantitative studies to examine how they informed each other.

## RESULTS

### Search Outcome and Study Characteristics

**Figure 1:**
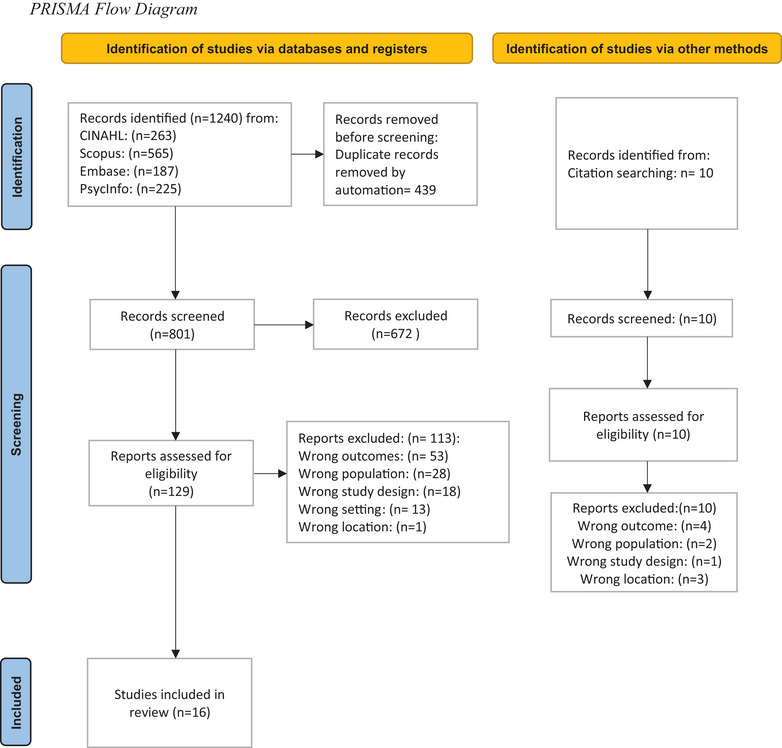
Preferred Reporting Items for Systematic Reviews and Meta‐Analyses Flow Diagram Outline of the process of identifying, screening, and selecting articles for the integrative review.

The search strategy generated 801 citations after removing duplicates (Figure 1). After reviewing titles and abstracts, 129 studies were examined by full text. A total of 16 studies published over 27 years (1996‐2022) were included in this review.^9^, ^10^, ^23^, ^24^, ^25^, ^26^, ^27^, ^28^, ^29^, ^30^, ^31^, ^32^, ^33^, ^34^, ^35^, ^36^ Of these, 2 were quantitative,^27^, ^35^ 13 were qualitative,^9^, ^10^, ^23^, ^24^, ^25^, ^26^, ^28^, ^29^, ^30^, ^31^, ^32^, ^33^, ^36^ and 1 was mixed method.^34^ Study characteristics and findings are displayed in Table 1. All 16 studies focused on exploring the health care experiences of Black birthing people,^9^, ^10^, ^23^, ^24^, ^25^, ^26^, ^27^, ^28^, ^29^, ^30^, ^31^, ^32^, ^33^, ^34^, ^35^, ^36^ 9 studies focusing on care during pregnancy only,^9^, ^23^, ^25^, ^26^, ^28^, ^29^, ^31^, ^33^, ^35^ and 7 studies,^10^, ^24^, ^27^, ^30^, ^32^, ^34^, ^36^ conducted during pregnancy and postpartum. Per the exclusion criteria, no studies were included from the intrapartum period. Although 3 studies^9^, ^23^, ^24^ recruited participants during the postpartum period, data were reported on both pregnancy and postpartum care experiences.

**Table 1:** Study Characteristics^a^

Author (Year) Region	Framework	Methodology	Sample Size	Black Birthing People Represented in Each Study, %	Period Reported On	Quality Rating
**Qualitative studies^a^**						
Adebayo et al^36^ (2022) Midwest	Critical race theory	Semistructured interviews	31	100	Pregnancy and postpartum	10/10
Kalata et al^28^ (2022) West	None	Focus groups	27	100	Pregnancy	7/10
Gross et al^24^ (2017) South	Positive deviance	Semistructured interviews	11	100	Pregnancy and postpartum	9/10
Mazul et al^31^ (2017) Midwest	None	Focus groups	31	100	Pregnancy	7/10
Roman et al^10^ (2017) Midwest	None	Focus groups	21	91	Pregnancy and postpartum	7/10
Lutenbacher et al^32^ (2016) not reported	Content analysis approach	Focus groups, semistructured individual interview	16	100	Pregnancy and postpartum	7/10
Tucker Edmonds et al^25^ (2015) Northeast	Community‐based participatory research	Focus groups	22	100	Pregnancy	6/10
Salm Ward et al^9^ (2013) Midwest	Jones theoretical framework for racism	Focus groups and individual interview	29	100	Pregnancy	8/10
Brubaker^33^ (2007) Not reported	Feminist critique of medicalization of reproduction	Semistructured interviews	51	100	Pregnancy	9/10
Cricco‐Lizza^29^ (2005) Northeast	Ethnography	Semistructured and unstructured interviews	11	100	Pregnancy	8/10
Sawyer^23^ (1999) West	Grounded theory	Focus groups and individual semistructure interview	17	100	Pregnancy	9/10
McAllister et al^26^ (1998) Not reported	None	Interview	13	100	Pregnancy	7/10
Murrell et al^30^ (1996) Not reported	Ethnography	Interviews	14	100	Pregnancy and postpartum	8/10
**Quantitative studies^b^**						
Almanza et al^27^ (2022) Midwest	Public health critical race praxis	Survey	314	13.2	Pregnancy and postpartum	7/14
Dahlem et al^35^ (2015) Midwest	Interaction model of client health behavior	Survey	204	100	Pregnancy	8/14
**Mixed‐methods studies^c^**						
Bentley et al^34^ (1999) South	None Ethnographic approach	Survey Unstructured interviews	441 80	100	Pregnancy and postpartum	3/5

Appraised using the JBI Critical Appraisal for Qualitative Studies.

Appraised using the NIH Quality Assessment for Cohort/Cross‐Sectional Studies.

Appraised using the Mixed‐Methods Appraisal Tool, Version 2018.

a Of note, no intrapartum care experiences were included.

Among the studies with a qualitative design, 6 used semistructured one‐on‐one interviews.^23^, ^24^, ^29^, ^32^, ^33^, ^36^ Whereas 9 used focus groups and interviews,^9^, ^10^, ^23^, ^25^, ^26^, ^28^, ^30^, ^31^, ^32^ 4 used focus groups only.^10^, ^25^, ^28^, ^31^ All quantitative studies used questionnaires for data collection. A meta‐analysis for quantitative articles could not be completed due to data insufficiency.

### Quality Appraisal

Seven qualitative studies had a low risk of bias, demonstrating good methodological quality (Table 1). Six qualitative studies showed moderate bias,^9^, ^23^, ^24^, ^29^, ^30^, ^33^, ^36^ with 4 not providing a philosophical framework.^10^, ^26^, ^28^, ^31^ Our interpretation for question 9 of the JBI quality assessment tool was if the description of the ethical approval process was not reported, this quality indicator was coded as a no. The mixed‐method study demonstrated a moderate risk of bias, with a score of 0.6.^34^ Inconsistencies between the qualitative and quantitative studies were not addressed, nor did the study adhere to the quality criteria of each methodological method involved. The quantitative articles showed a high and moderate risk of bias, with quantitative critical appraisal scores falling between 0.5 and 0.57.^27^, ^35^ The weak aspects of the quantitative studies were related to methodological issues, such as clarity of sampling strategy (lack of inclusion of any missing data or power analysis required for sample size) or data collection and analysis methods (regarding failure to include the measure's psychometric properties).

### Outcomes Reported

**Figure 2:**
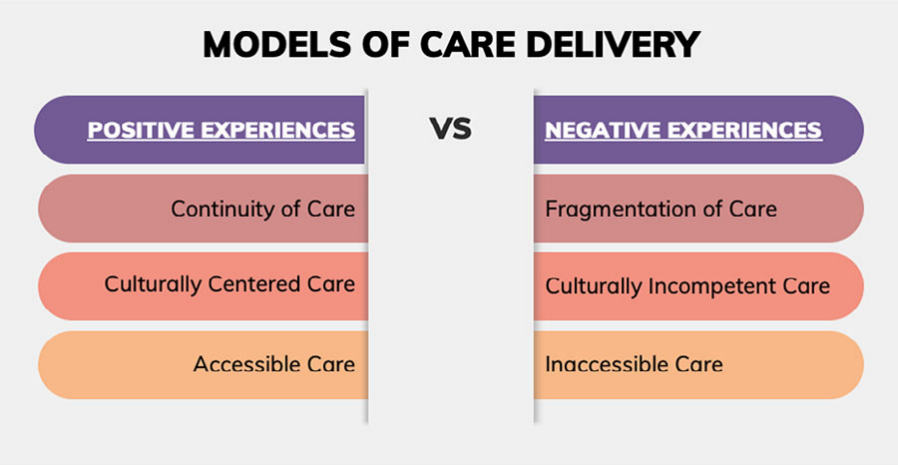
Models of Care Delivery Models of care delivery that contributed to positive and negative health care experiences.

**Figure 3:**
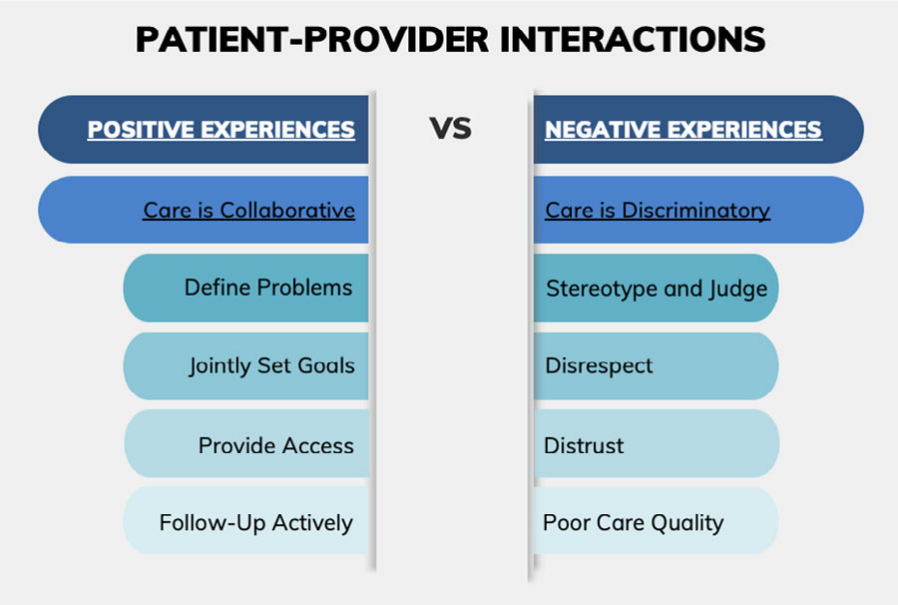
Patient‐Provider Interactions Patient‐provider interactions that contributed to positive and negative health care experiences.

A dichotomization of positive and negative health care experiences became apparent when examining the studies. As such, the findings are reported to reflect care experiences that were attributed to as either positive or negative. Furthermore, after analyzing the study themes, 2 global themes became apparent: models of care delivery (Figure 2) and patient‐provider interactions (Figure 3). These 2 global themes encompassed care experiences that were both positive and negative, with the factors that contributed to each being consistent during the pregnancy and postpartum reporting. Please refer to Table 2 for a detailed description of the themes identified in each study.

**Table 2:** Themes Identified in the Included Qualitative Studies

First Author	Themes Identified
Adebayo et al^36^ (2022)	4 themes identified: Institutionalized care—racially insensitive biomedical approach Race and class—unfair treatment based on health insurance^a^ Race and social concept—dismissed pain concerns because you are a strong Black woman^a^ Distrust—African American women as a charity case^a^
Kalata et al^28^ (2022)	6 themes identified: Meaning of healthy pregnancy Advice during pregnancy^a^ Barriers to prenatal care^a^ Type of prenatal care received^a^ Comfort voicing concerns during pregnancy^a^ Barriers to social and emotional support^a^
Gross et al^24^ (2017)	4 themes identified: Deciding to breastfeed^a^ (subthemes: point of decision, family influence, costs, health benefits,^a^ birth order) Initiating breastfeeding^a^ (subthemes: birth and hospital experience,^a^ WIC peer counselors, pumping) Breastfeeding long‐term (subthemes: developing a routine, returning to work, social support, infant bonding, strategies) Expanding breastfeeding support
Mazul et al^31^ (2017)	4 themes identified: Structural barriers^a^ Psychosocial barriers Attitudes and perceptions^a^ Facilitators^a^
Roman et al^10^ (2017)	4 basic themes identified: Pursuit of prenatal care Experiences of traditional prenatal care^a^ Enhanced prenatal and postpartum care^a^ Women's health: a missed opportunity^a^ 2 global themes identified: Communication with providers^a^ Perceived socioeconomic or racial bias^a^
Lutenbacher et al^32^ (2016)	4 themes identified: Balancing the influences: people, myths, and technology^a^ (subthemes: role models: my circle of friends and family, my personal tug of war, Google as my friend^a^) Being in the know Critical periods^a^ (subthemes: prenatal, early postpartum,^a^ returning to work or school) Supportive transitions
Tucker Edmonds et al^25^ (2015)	2 themes identified: Creating a perfect prenatal care system: dislike/change (subthemes: lack of personal relationship/connection, discontinuity,^a^ trainees and medical students, lack of care and compassion,^a^ lack of respect, judgment or stereotyping, wait times) Creating a perfect prenatal care system: keep/like (subthemes: midwifery model,^a^ ultrasound, nursing and staff continuity^a^)
Salm Ward et al^9^ (2013)	3 themes identified: Discrimination based on insurance or income status^a^ Discrimination based on race during prenatal care^a^ Discrimination based on race over their lifetime
Brubaker^33^ (2007)	3 themes identified: Social problems discourse denies teens knowledge and access to formal care Embracing medicalization^a^ Resisting medicalization^a^
Cricco‐Lizza^29^ (2005)	No themes identified The WIC clinic promoted knowledge and positive breastfeeding messaging through use of multiethnic posters. Lactation consultants provided information, assistance, and recommendations that was considered helpful during care
Sawyer^23^ (1999)	Core theme identified: engaged mothering^a^
McAllister and Boyle^26^ (1998)	4 themes identified: Wanting to be away Makin’ it Being alone Receiving care^a^
Murrell et al^30^ (1996)	3 themes identified: The pervasiveness of the stereotypes of pregnant African American women^a^ Care that is indifferent, inaccessible, and undignified^a^ The totality of racism
Bentley et al^34^ (1999)	Ethnographic results: Women were affected positively or negatively by physicians or other health care professionals regarding their newborn feeding decisions

Abbreviation: WIC, Special Supplemental Nutrition Program for Women, Infants, and Children.

a Denotes theme that was included in study.

### Models of Care Delivery

Five studies reported on models of care delivery that contributed to a positive care experience.^10^, ^25^, ^26^, ^27^, ^28^, ^29^ These models promoted continuity of care, culturally centered care, or accessible care. Nine studies reported on models of care delivery that contributed to a negative care experience.^9^, ^10^, ^23^, ^25^, ^28^, ^29^, ^30^, ^31^, ^32^ These models incorporated fragmented care, culturally insensitive care, or inaccessible care.

### Positive Care Experience

Continuity of care, described in 2 studies^25^, ^26^ led to a positive care experience between the participants and their health care team. For example, Tucker Edmonds et al^25^ discovered that participants formed bonds with nurses and office staff, making them feel cared for as if they were family members.^25^ McAllister and Boyle^26^ identified that participants viewed continuity of care as receiving “first‐class care,” which was not typical for many Medicaid recipients.

Next, 4 studies^10^, ^27^, ^28^, ^29^ identified culturally centered care, in which the provider shares similar experiences or racial background with the participant, led to a more positive experience. Roman et al^10^ found that during prenatal care, birthing people felt that having community health workers who they could relate to and identify as “someone like me” was important for navigating challenging situations.^10^ Kalata et al^28^ found that some participants trusted their provider more if they were racially congruent, making them feel more comfortable and less defensive.^28^ Lastly, Almanza et al^27^ found that participants who received care from a birth center that promoted a culturally centered model experienced statistically higher levels of perceived autonomy and respect, regardless of race.^27^ Finally, one study^26^ identified that accessible care, which was characterized by prenatal care within walking distance from home and the availability of insurance coverage, contributed to a positive care experience. McAllister and Boyle^26^ found that accessible care (all clients had the same insurance) contributed to care provided without stigma.

### Negative Care Experience

Consequently, fragmented care, typified by seeing a new provider at each visit or repeating the same information to a provider at every visit, was identified in 3 studies^10^, ^25^, ^28^ and contributed to a negative care experience. Roman et al^10^ described the energy birthing people expend repeating their information to providers, often wondering if they are being heard. Kalata et al^28^ found participants felt defensive and exhausted as they continuously reintroduced or re‐explained decisions to providers related to their prenatal care. Tucker Edmonds et al^25^ noted participants’ frustration at being a “paper patient,” referring to the brief chart audits that providers conduct before providing prenatal care but without the establishment of a meaningful patient‐provider relationship.

The importance of culturally sensitive care was identified in 3 studies,^23^, ^28^, ^29^ which found that providers who lacked awareness in the diversity of Black birthing people's values or used material seen as culturally inappropriate led to negative care experiences. Kalata et al^28^ explored how implicit bias influenced the birth options discussed with individuals during prenatal care, leaving birthing people feeling like they lack autonomy in decision‐making. Additionally, Sawyer^23^ examined how participants felt *othered* through pregnancy care. One participant reported that during a childbirth class the instructor asked the participant to describe “what would you do in your culture” because they were the only African American students. Cricco‐Lizza^29^ described the adverse reactions participants had when viewing nutritional educational videotapes compared with the positive experience of having culturally sensitive lecture‐discussion sessions with a nutritionist.

Finally, social determinants of health, such as lack of insurance, transportation difficulties, location of offices, or limited office hours, were identified in 6 studies^9^, ^10^, ^28^, ^30^, ^31^, ^32^ as factors contributing to inaccessible care, thus fostering a negative health care experience. As an exemplar, Mazul et al^31^ illustrated how participants either had to wait for insurance coverage to be seen or had to change providers during the pregnancy because their insurance was no longer accepted. Lastly, 2 studies^28^, ^31^ identified limited appointment hours or the location of offices as a hindrance in receiving care.

### Patient‐Provider Interactions

Eleven studies reported positive patient‐provider interactions, defined by participants as collaborative care, defining problems, jointly setting goals, providing access, and actively following up.^10^, ^23^, ^24^, ^25^, ^26^, ^29^, ^31^, ^32^, ^33^, ^34^, ^35^ Ten studies reported negative patient‐provider interactions involving discriminatory, stereotypical, judgmental, disrespectful, distrustful, or poor‐quality care.^9^, ^10^, ^23^, ^25^, ^28^, ^30^, ^31^, ^32^, ^33^, ^36^

### Positive Care Experience

A collaborative care environment was emphasized in 7 studies^10^, ^25^, ^29^, ^31^, ^32^, ^33^ centering on patient‐provider interactions that resulted in participants experiencing high levels of autonomy and feeling cared for and valued. Brubaker^33^ noted the importance of positive attention from providers, often making participants feel like they were part of the family. A provider's capacity to make participants feel valued, whether through acts of kindness or by taking time to know each participant, further contributed to nurturing a collaborative environment between patients and clinicians.^26^, ^31^ Finally, Tucker Edmonds et al^25^ noted that participants felt more engaged and empowered and had a greater sense of autonomy when receiving care provided by midwives.

Next, problems that are well defined during interactions, as explained by Cricco‐Lizza,^29^ particularly concerning breastfeeding support, contributed to positive patient‐provider interactions. Additionally, jointly setting goals between providers and patients during breastfeeding interactions, as delineated in 3 studies,^24^, ^32^, ^34^ was identified as being beneficial. For example, Gross et al discussed the importance of goal setting through provider‐led discussions on breastfeeding over the course of pregnancy.

The accessibility of providers, emphasized in 4 studies,^23^, ^24^, ^33^, ^35^ fostered increased levels of trust and comfort during patient‐provider interactions. For example, when examining patient‐provider communication and trust, Dahlem et al^35^ found that higher patient‐provider communication scores significantly increased trust in providers and overall patient satisfaction. Sawyer^23^ incorporated a participant's level of comfort in their provider finding that “comfort with” was significant in selecting a provider. Finally, actively following up, such as making a phone call after missing an appointment, was discovered to improve patient‐provider interactions.

### Negative Care Experience

The description of the negative care experiences was very specific, with most studies identifying specific examples of racial discrimination affecting patient‐provider interactions. Participants described experiences of discriminatory care in 9 studies,^9^, ^10^, ^23^, ^28^, ^30^, ^31^, ^32^, ^33^, ^36^ in which they felt that their race, socioeconomic status, or both contributed to differences in the quality of care received and resulted in an overall negative care experience. Salm Ward et al^9^ described how a provider's negative assumption about Black birthing people (assuming medical assistance, unmarried, or use of drugs) negatively influenced the prenatal care experience, giving the example of a participant being asked if they were on crack because of their race. Roman et al^10^ explored participant experiences of discrimination during prenatal and postpartum care and found both dismissive and nonverbal actions contributed to negative interactions and perceptions of racism, especially concerning the topic of postpartum contraception.

Moreover, participants reported feeling stereotyped or judged in their interactions with health care providers, described in 6 studies.^9^, ^10^, ^23^, ^30^, ^32^, ^36^ Providers were noted to make assumptions about income, education, pain levels, or marital status based on race. Adebayo et al^36^ conveyed how Black birthing people are judged or receive different care relating to pain based on race. Roman et al^10^ found that participants felt as though they were not listened to by health care professionals, and explored how detrimental provider stereotypes (regarding race and income) influenced the care experience, with participants often being made to feel incompetent or uneducated because of social factors.

Disrespectful patient‐provider interactions, noted in 3 studies,^25^, ^28^, ^33^ arose from verbal and nonverbal communication between patient and provider. Brubaker^33^ described the voices of participants who encountered disrespectful interactions with health care providers, often stemming from racial biases. Kalata^28^ observed that participants encountered disrespectful tones characterized as patronizing or demeaning, which resulted in feelings of experiencing discriminatory care.

Additionally, distrust in providers, described in 4 studies,^10^, ^28^, ^30^, ^36^ contributed to a fear of sharing information or reluctance to ask for help when needed. Adebayo et al^36^ described Black birthing peoples’ perception of how their racial identity positioned them as a charity case and led them to have high levels of distrust with their health care providers. Kalata et al^28^ explored how generations of distrust in the health care system due to mistreatment and unethical care among African Americans promoted the fear of sharing essential information with health care team members. Finally, Roman et al^10^ examined participants’ reluctance to share information with their providers out of fear of potential negative repercussions.

Lastly, perceptions of poor‐quality care as described in 4 studies^10^, ^28^, ^30^, ^31^ were associated with negative experiences with care. Mazul et al^31^ found that participants did not find value in attending prenatal care appointments due to feeling rushed, dismissed, or uncared for during previous visits. Roman et al^10^ found that the importance of a postpartum visit was not explained to many Black birthing people. Finally, Murrell^30^ found critical differences in perceptions of care delivery between Black and White birthing people. The differences were frequently attributed to factors such as race, socioeconomic status, or insurance type.

## DISCUSSION

Our integrative review summarizes published evidence on the diverse prenatal and postpartum care experiences among Black birthing people in the United States. Three significant findings from this review were found to affect the experience of care among Black birthing people, including models of care delivery, patient‐provider interaction, and lack of focus on postpartum care.

Models of care were a major contributor to positive care experiences. The standard prenatal care model focuses on individualized visits.^37^ However, alternative models exist, varying between group prenatal care or birth centers. Group prenatal care, such as Centering Pregnancy, provides continuity of prenatal care, education, and peer support to promote health and wellness and optimize birth outcomes. Group prenatal care is associated with a 3% decreased risk of preterm birth among Black birthing people.^38^ However, research has focused more on newborn outcomes instead of perinatal outcomes.^39^ Further research is needed to examine whether group prenatal care decreases racial disparities and improves severe maternal morbidity and mortality among Black birthing people.^39^

The birth center model of care involves freestanding birth centers usually directed by midwives who offer comprehensive midwifery care in a home‐like setting.^40^, ^41^ The birth center model emphasizes education, psychosocial support, and low rates of medical intervention.^40^ Roots Community Birth Center as described by Almanza et al^27^ has successfully addressed racial inequities by promoting continuity and culturally centered care. Roots improves postpartum care by integrating dyadic mother‐child appointments, incorporating 7 visits within the first 6 weeks to assess care needs. The overall care experience is grounded in respect for the birthing person's culture, making patients feel respected, valued, and heard.^41^

The interactions between patient and providers plays a crucial role in influencing patients’ care experiences, which in turn affects overall health care outcomes.^42^ Patient‐provider interactions among Black birthing people were extensively intertwined with care experiences marked by discrimination and disrespect, which contributed to the perception of poor‐quality care. Furthermore, Altman et al^42^ found that unequal power dynamics within the care environment contributes to an overall negative health care experience. The belief that the “provider knows best” has been perpetuated in health care communities, affecting how health care information is packaged and delivered to patients.^42^, ^43^ The manner in which information is presented is then informed by the relationship with the patient, which factors in any stereotypes, judgments, and biases.^42^ Future research may consider examining how providers’ implicit biases, stereotypes, and judgments influence the provision of health care information and effect on health care outcomes among Black birthing people.

Race and socioeconomic status‐based discrimination is experienced at systemic, institutional, and interpersonal levels, with more than half of the studies in this review mentioning interpersonal experiences of discrimination among Black birthing people during their prenatal or postpartum care. Discrimination is the differential treatment of others based on race, socioeconomic status, gender, ethnicity, disability, religion, nationality, or geographic location.^44^, ^45^ Black birthing people encounter higher levels of racial discrimination in comparison with White birthing people.^46^ Interpersonal experiences of racial discrimination is a psychosocial stressor that is associated with poor health outcomes including hypertension and cardiovascular disease among Black birthing people.^47^, ^48^ However, evidence on the impact of health care discrimination is lacking. Further research is needed to examine how discrimination in health care settings influences health disparities in the prenatal and postpartum care settings.

Finally, more than half (53%) of pregnancy‐related deaths occur between one week and one year postpartum, yet no studies in this review focused solely on care during the postpartum period.^49^ Recently, more attention has been given to increasing access to postpartum care through Medicaid expansion and updating professional guidelines.^50^ However, despite these measures, Black birthing people remain less likely to attend postpartum visits when compared to non‐Black birthing people.^50^ Roman et al^10^ found that participants who skipped their postpartum visit did so because they either experienced poor‐quality prenatal care or the importance of the postpartum visit was not stressed during pregnancy. Further research is needed to explore barriers and facilitators for postpartum care among Black birthing people. Additionally, research is needed to assess the care experience and what policymakers, health care systems, and health professionals can do to address the gaps identified during this critical period.

### Strengths and Limitations

We included quantitative, qualitative, and mixed‐method studies, used validated quality assessment tools, and incorporated multiple reviewers to decrease selection bias. The consolidation of qualitative and quantitative data from multiple studies provides a more in‐depth understanding of the interactions, attitudes, and beliefs among Black birthing people within prenatal and postpartum care. The results from this study provide a more comprehensive basis for complex decision‐making than single‐method reviews, thus increasing benefit to policymakers and clinicians.^21^ We recognize several limitations. Half of the studies are greater than 10 years old; however, the same findings reported 30 years ago are still being cited in the most recent articles. Furthermore, despite findings that spanned 3 decades of research, there was a lack of community‐based participatory action research, a limited number of quantitative studies, and no randomized control trials of the effectiveness of models of care explicitly designed to address poor care experiences among Black birthing people. This hampers the ability to complete a meta‐analysis, limits the robustness of results, and highlights key opportunities in this area of science. For example, the field would greatly benefit from randomized control trials formally evaluating the ability of innovative models of care to address complex issues such as implicit bias, inequitable care interactions, and provider distrust. Such data are needed to ensure that the onus is placed on health care systems and health care providers, not patients. Furthermore, 4 of the included studies were identified as having a moderate risk of bias for methodological quality, which could compromise the validity of our findings. Finally, we recognize our interpretations are limited to our own life experiences and professional training as nurses, midwives, and nurse practitioners (with all researchers identifying as White).

### Implications for Practice

There are several ways midwives and other health care providers may address health inequities present in maternal morbidity and mortality among Black birthing people. Midwives and other health providers may consider increasing their self‐knowledge of implicit bias and be cognizant of verbal and nonverbal cues during patient interactions. Social determinants of health play a significant role in access to care, and clinicians may have limited ability to change their practice. However, midwives and other health care providers may help drive change by increasing available office hours for appointments, having a flexible late appointment policy, offering patients resources for transportation and childcare, and considering changes to the schedule and delivery of visits during the prenatal and postpartum period. Midwives and other health care providers may also advocate for and support policy changes that increase access to health insurance and health care. Finally, as discussed above, further research is needed to inform practice change and health care policy.

## CONCLUSIONS

Black birthing people in the United States report positive and negative health care experiences during pregnancy and postpartum care. Patients are clear on how clinicians, offices, and systems can both facilitate positive experiences and prevent negative experiences. The negative experiences may be contributing to known race‐ and ethnicity‐based health inequities. Health care models that promote continuity of care, culturally centered care, and collaborative patient‐provider relationships have been shown to contribute to positive health care experiences. More research is needed on the care experience during the postpartum period specifically. Promoting high‐quality, collaborative, and culturally centered prenatal and postpartum care is needed to foster patient safety and improve clinical outcomes.

## CONFLICTS OF INTEREST

The authors have no conflicts of interest to disclose.

## Supporting information




**Table S1**. PRISMA 2020 Checklist

**Table S2**. Search Strategy
