# Supplementary material for: Prenatal and Postpartum Care Experiences Among Black Birthing People in the United States: An Integrative Review
Source: J Midwifery Womens Health. 2024 Nov 13;70(2):235–46. doi: 10.1111/jmwh.13705 (PMC11980764; doi:10.1111/jmwh.13705)
Supplement: Supplementary file 2 — Table S2. Search Strategy [file JMWH-70-235-s002.docx]

**Supplemental Table 2.** Search Strategy

| Data Base | Search Strategy | Results |
| --- | --- | --- |
| CINAHL | (prenatal OR pre-natal OR perinatal OR peri-natal OR postpartum OR post-partum)/care/African American OR Black American OR Blacks/Experience) | 263 |
| Scopus | ((prenatal  OR  perinatal  OR  pre-natal  OR  postpartum  OR  post-partum/care/  "African American"  OR  "Black American"  OR  blacks/experience*)) AND (LIMIT-TO ( LANGUAGE ,  "English")) | 565 |
| Embase | ('prenatal' OR 'prenatal'/exp OR prenatal OR perinatal OR 'pre natal' OR 'postpartum' OR 'postpartum'/exp OR postpartum OR 'post partum') AND ('care' OR 'care'/exp OR care) AND ('african american'/exp OR 'african american' OR 'black american'/exp OR 'black american' OR 'blacks' OR 'blacks'/exp OR blacks) AND ('experience' OR 'experience'/exp OR experience) AND [english]/lim AND [humans]/lim | 187 |
| PsycInfo | ( prenatal OR pre-natal OR perinatal OR peri-natal OR postpartum OR post-partum) ) AND care AND ( african americans or black americans or blacks ) AND experience | 225 |
